# Relationship Between Neck Circumference and Epicardial Fat Thickness in a Healthy Male Population

**DOI:** 10.5935/abc.20160112

**Published:** 2016-09

**Authors:** Uğur Küçük, Hilal Olgun Küçük, Ferhat Cüce, Sevket Balta

**Affiliations:** 1 Gulhane Military Medical Academy Haydarpasa Training Hospital, Ankara - Turkey; 2 Department of Cardiology, İstanbul, Dr. Siyami Ersek Thoracic and Cardiovascular Surgery Center Training and Research Hospital, Ankara - Turkey; 3 Department of Cardiology, İstanbul, Van Army District Hospital, Ankara - Turkey; 4 Department of Radiology, Van, Gulhane Military Medical Academy Department of Cardiology, Ankara - Turkey

**Keywords:** Neck, Intra-Abdominal Fat, Blood Pressure, Cardiovascular Diseases, Body Mass Index, Blood Glucose, Echocardiography / diagnosis

## Abstract

**Background:**

Epicardial fat is an upper body visceral fat depot that may play a significant role in the development of adverse metabolic and cardiovascular risk profiles. There is a significant direct relationship between the amount of epicardial fat and general body adiposity (body mass index, BMI), but data regarding subcutaneous adiposity is limited.

**Objective:**

We conducted a study to determine the association between neck circumference and epicardial fat thickness in healthy young male individuals, and assess their individual correlations with general body adiposity and cardiometabolic risk factors.

**Methods:**

One hundred consecutive male patients aged 18 years or older with no known major medical conditions were included in the study. All participants underwent detailed physical examination including measurement of blood pressure, weight, height, waist/hip ratio, and neck circumference. Blood was collected to determine fasting glucose and lipid parameters. A standard echocardiographic examination was performed with additional epicardial fat thickness determination.

**Results:**

Among 100 study participants, neck circumference correlated significantly with weight, waist circumference, BMI, blood glucose, serum total cholesterol, low-density (LDL)-cholesterol, and triglycerides levels. No significant correlation was found between neck circumference and high-density lipoprotein (HDL)-cholesterol levels. Neck circumference correlated moderately and positively with echocardiographic epicardial fat thickness.

**Conclusion:**

Among patients with low cardiometabolic risk, increased neck circumference was associated with increased epicardial fat thickness.

## Introduction

During the past 20 years, numerous discoveries dramatically changed our view of the adipose tissue from a simple storage depot to an active endocrine organ. In addition to its major role in lipid and glucose metabolism, the adipose tissue participates in the signaling of systemic homeostasis. The two major types of adipose tissue are visceral fat, localized within the abdominal cavity and mediastinum, and subcutaneous fat, localized in the hypodermis.

Neck circumference, a proxy for upper body subcutaneous fat, is a unique fat depot that confers additional cardiovascular risk above and beyond central body fat.^1^ Epicardial fat is an upper body visceral fat depot that may play a significant role in the development of adverse metabolic and cardiovascular risk profiles. It modulates local functions of the coronary artery and is further implicated in the pathogenesis of coronary artery disease.^2,3^

However, no studies have examined the association between neck circumference and epicardial fat. Thus, the goal of this analysis was to characterize the correlation between neck circumference and epicardial fat and answer the following specific question: is increased neck circumference associated with increased epicardial fat thickness in healthy male subjects with low cardiometabolic risk?

## Method

We recruited 100 consecutive male patients aged 18 years or older without known major medical conditions (*e.g.* , diabetes, coronary artery disease, hypertension, or thyroid or malignant diseases) and not receiving prescription medication. All subjects had attended annual periodic health examinations between November 2013 and May 2013. The participants were informed about the study procedures and agreed to participate providing written informed consent.

All measurements were performed by one investigator using the following standard techniques: weight, measured on a scale (Holtain, Wales) to the nearest 100 g with the participant wearing light clothing; height, measured with a portable stadiometer with the participant barefoot (Holtain, Wales) to the nearest 0.5 cm; and waist and hip circumferences, measured with weekly calibrated plastic tapes to the nearest 1 mm. The waist circumference was measured at the end of gentle expiration midway between the lowest rib and the iliac crest with the patient standing, while the hip circumference was measured at the greater trochanter.^1^ Body mass index (BMI) was calculated as weight in kilograms divided by the square of the height in meters. Neck circumference was measured to 1-mm accuracy with a plastic tape in a standardized manner, horizontally above the cricothyroid cartilage, just below the laryngeal prominence.^4^ All measurements were taken with the subjects standing upright, facing the investigator, and with shoulders relaxed.

Systolic and diastolic blood pressure was measured twice in all participants by the same physician using a standard aneroid sphygmomanometer on the right arm of the seated subject. The mean value for blood pressure measurements was adopted. After a 12-hour fasting, blood samples were collected for analyses of blood glucose, total cholesterol, HDL-cholesterol, and triglycerides. Epicardial fat was assessed via transthoracic echocardiography (ProSound 6, Hitachi-Aloka, Tokyo Japan). A standard echocardiographic examination was performed in all participants. Maximum epicardial fat thickness was measured from a two-dimensional long-axis view on the right ventricular free wall perpendicular to the aortic annulus or at a mid-chordal level from a parasternal short-axis view at the tip of the papillary muscle at end-systole. Average values of three cardiac cycles from each echocardiographic view were determined. Based on previous studies, the upper normal limit for epicardial fat thickness was determined as 7 mm.^5^

### Statistical analysis

Continuous variables are expressed as mean ± standard deviation (SD). All statistical calculations were performed using SPSS 18 (SPSS Inc., Chicago, IL, USA). Normality was tested using the Kolmogorov-Smirnov test in addition to graphical methods (probability-probability plots and histograms). As both parameters were normally distributed, the correlation coefficients and their significance were calculated using Pearson test. Neck circumference and epicardial fat measurements were divided into five equal groups, and intraobserver variability was investigated using the Kappa test. A multiple regression model was used to identify independent predictors of epicardial fat thickness. The model fit was assessed using appropriate residual and goodness of fit statistics. A 5% type-I error level was used to infer statistical significance.

## Results

**Figure 1:**
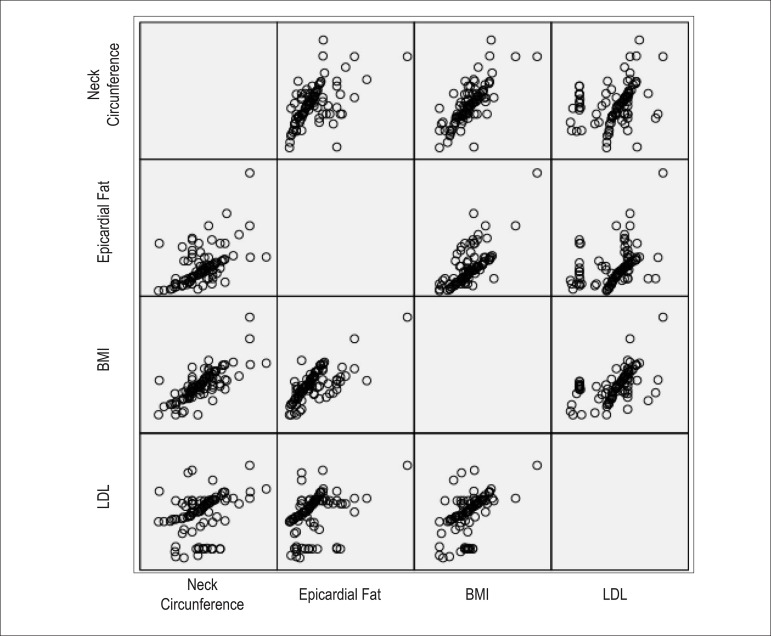
- Matrix scatterplot showing associations between neck circumference, epicardial fat, BMI, and LDL-cholesterol.

The study sample consisted of 100 male individuals with a mean age of 26.0 ± 4.3 years. None of the patients had documented major comorbidities. The mean BMI of the participants was 24.9 ± 3.5 kg/m^2^ and the mean neck circumference was 39.4 ± 2.39 cm (Table 1). In correlation analysis among all subjects, neck circumference correlated significantly with weight, waist circumference, and BMI, and moderately with serum total cholesterol, LDL-cholesterol, and triglycerides levels. No significant correlation was found between neck circumference and HDL-cholesterol levels (Figure 1). Neck circumference correlated moderately and positively with echocardiographic epicardial fat thickness. A matrix scatter plot in the Figure demonstrates a linear association between neck circumference, epicardial fat, BMI, and LDL-cholesterol. We used multiple regression analysis to test if the neck circumference predicted significantly the epicardial fat thickness. The results indicated that neck circumference, BMI, and LDL-cholesterol explained 79% of the variance (R2 = 0.799, F[3,13] = 17.2, p < 0.01). We found that neck circumference significantly predicted epicardial fat thickness (β = 0.879, p < 0.001). We also observed a good intraobserver agreement for neck circumference and epicardial fat measurements (Kappa values = 0.723 and 0.715, respectively, p values = 0.574 and 0.974, respectively).

**Table 1:** Association between neck circumference and clinical, laboratory and echocardiographic parameters

	Correlation coefficients		
		Neck Circumference	
	Mean ± SD	R*	p
Altura, cm	175 ± 7.32	0.111	0.272
Peso, kg	76.7 ± 10.87	0.715	< 0.001
IMC, kg/m^2^	24.9 ± 3.50	0.673	< 0.001
Cintura, cm	90 ± 9.54	0.638	< 0.001
Quadril, cm	103 ± 7.2	0.191	0.06
Colesterol total, mg/dL	183 ± 35.86	0.435	< 0.001
Triglicerídeos, mg/dL	173 ± 54.9	0.338	< 0.001
LDL-colesterol, mg/dL	83.9 ± 25.84	0.432	0.014
HDL-colesterol, mg/dL	45.5 ± 9.59	0.201	0.271
Gordura epicárdica, mm	2.98 ± 1.26	0.474	< 0.001

SD: standard deviation; BMI: body mass index; LDL: low-density lipoprotein; HDL: high-density lipoprotein; R: Pearson correlation coefficient

## Discussion

This study indicates a correlation between neck circumference and epicardial fat thickness, as well as between neck circumference and other anthropometric measures in healthy, nonobese male individuals. Neck circumference also showed a strong correlation with serum total cholesterol, LDL-cholesterol, and triglyceride levels.

The distribution of body adiposity is a stronger predictor of metabolic dysfunction and cardiovascular risk than whole-body adiposity, which is measured with the BMI.^6^ The wide use of the waist circumference relies on its correspondence to abdominal visceral fat, which is thought to have a major role in cardiometabolic risk.^7^ Apart from waist circumference, other circumferences have also been evaluated as anthropometric indices, including neck, hip, thigh, arm, and calf circumferences. Among them, neck circumference is an alternative measure of upper body subcutaneous fat, which relates to cardiometabolic risk as much as abdominal visceral adipose tissue (VAT).^8^ Consistent with previous reports,^9^ this study showed that neck circumference correlated well with waist circumference, waist-to-hip ratio, and BMI. Compared with waist circumference, neck circumference is easier to measure and has low intra- and interobserver variability.^10^

In the Framingham Heart Study, neck circumference was associated with cardiometabolic risk factors even after adjustment for VAT.^8^ Similarly, we have shown a positive correlation between neck circumference, serum total cholesterol, LDL-cholesterol, and triglycerides levels. Based on these findings, some authors have suggested the use of neck circumference as a tool for identification of metabolic syndrome and insulin resistance.^9^ These correlations transform further into the clinical picture with numerous data reporting associations between clinical/subclinical atherosclerosis and neck circumference.^11-13^

Epicardial fat is located on the surface of the heart especially around the epicardial coronary vessels. It is the true visceral fat depot of the heart. Under normal physiological conditions, epicardial fat has several putative functions: it protects the heart against excessively high circulating levels of fatty acids, acts as a local energy source at times of high demand channeling fatty acids to the myocardium, and buffers the coronary arteries against the torsion induced by the arterial pulse wave and cardiac contraction.^14,15^ Epicardial fat is also a source of several proinflammatory and proatherogenic cytokines, as well as tumor necrosis factor-α, monocyte chemoattractant protein-1, interleukin-6, leptin, plasminogen activator inhibitor-1, and angiotensinogen.^16,17^ Epicardial fat also produces antiinflammatory and antiatherogenic adipokines, such as adiponectin and adrenomedullin.^5,18^ In general, epicardial fat exerts a protective modulation of vascular function and energy partition in a healthy situation, but when expanded, it turns into an adverse lipotoxic, prothrombotic, and proinflammatory organ.^19,20^

Epicardial fat thickness can be visualized and measured with two-dimensional echocardiography, magnetic resonance imaging, and/or computed tomography. On echocardiography, epicardial fat thickness clearly reflects visceral adiposity and increases with an increase in general adiposity. In hearts with markedly increased epicardial fat mass, epicardial fat thickness shows a highly significant correlation with body weight.^21^ Autopsy studies, however, report a weak correlation between BMI and epicardial fat. Several autopsy studies have evaluated the correlation between epicardial fat and subcutaneous adipose tissue. Womack et al. reported a significant correlation between epicardial fat and the total amount of fat in the calf in both sexes.^22^ Besides all above mentioned associations between various subcutaneous fat tissues and epicardial fat, there is a paucity of studies relating neck circumference to epicardial fat as a proxy of upper body subcutaneous adiposity. This is the first study demonstrating a significant correlation between neck circumference and epicardial fat thickness.

Considering that echocardiographic epicardial fat thickness correlates with metabolic syndrome, insulin resistance, coronary artery disease, and subclinical atherosclerosis, it might serve as a simple tool for cardiometabolic risk prediction.^23-25^ Substantial changes in echocardiographic epicardial fat thickness during weight loss may also suggest its use as a marker of therapeutic effect. However, the requirement of echocardiography to measure epicardial fat thickness limits its widespread use in clinical settings, whereas measurement of neck circumference is a simple, low cost, and informative tool that every healthcare provider can utilize in assessing cardiometabolic risk and estimating epicardial fat thickness.

## Conclusion

Neck circumference was a reliable and feasible alternative measurement that correlated well with other anthropometric measurements and cardiometabolic parameters. It is also strongly associated with epicardial fat thickness. We suggest neck circumference measurement to be used to estimate epicardial fat thickness during daily clinic practice.

### Limitations

Since this study included only healthy young men, we are unable to determine if the results could be applied to other populations including women and individuals with metabolic syndrome or other comorbidities. All measurements were performed by the same author, which makes the study prone to systematic error.
